# Quaking Deficiency Amplifies Inflammation in Experimental Endotoxemia *via* the Aryl Hydrocarbon Receptor/Signal Transducer and Activator of Transcription 1–NF-κB Pathway

**DOI:** 10.3389/fimmu.2017.01754

**Published:** 2017-12-08

**Authors:** Li Wang, Dong-Sheng Zhai, Ban-Jun Ruan, Cheng-Ming Xu, Zi-Chen Ye, Huan-Yu Lu, Ying-Hao Jiang, Zhen-Yu Wang, An Xiang, Yuan Yang, Jian-Lin Yuan, Zi-Fan Lu

**Affiliations:** ^1^State Key Laboratory of Cancer Biology, Department of Pharmacogenomics, School of Pharmacy, Fourth Military Medical University, Xi’an, China; ^2^Department of Occupational and Environmental Health and the Ministry of Education Key Laboratory of Hazard Assessment and Control in Special Operational Environment, School of Public Health, Fourth Military Medical University, Xi’an, China; ^3^Department of Urology, Xijing Hospital, Fourth Military Medical University, Xi’an, China

**Keywords:** quaking, macrophage polarization, endotoxemia, aryl hydrocarbon receptor, NF-κB

## Abstract

Macrophages, characterized by considerable diversity and plasticity, play a crucial role in a broad spectrum of biological processes, including inflammation. However, the molecular mechanisms underlying the diverse phenotypes of macrophages are not well defined. Here, we show that the RNA-binding protein, quaking (QKI), dynamically modulates macrophage polarization states. After lipopolysaccharide (LPS) stimulation, QKI-silenced RAW 264.7 cells displayed a pro-inflammatory M1 phenotype characterized by increased expression of iNOS, TNF-α, and IL-6 and decreased expression of anti-inflammatory factors, such as IL-10, found in inflammatory zone (Fizz1), and chitinase-like 3 (Chil3 or Ym1). By contrast, QKI5 overexpression led to a suppressive phenotype resembling M2 macrophages, even under M1 differentiation conditions. Moreover, myeloid-specific QKI-deficient mice tended to be more susceptible to LPS-induced endotoxic shock, while the exogenous transfer of macrophages overexpressing QKI5 exerted a significant improving effect. This improvement corresponded to a higher proportion of M2 macrophages, in line with elevated levels of IL-10, and a decrease in levels of pro-inflammatory mediators, such as IL-6, TNF-α, and IL-1β. Further mechanistic studies disclosed that QKI was a potent inhibitor of the nuclear factor-kappa B (NF-κB) pathway, suppressing p65 expression and phosphorylation. Strikingly, reduced expression of the aryl hydrocarbon receptor (Ahr) and reduced phosphorylation of signal transducer and activator of transcription 1 in QKI-deficient cells failed to restrain the transcriptional activity of NF-κB and NRL pyrin domain containing 3 (NLRP3) activation, while restoring QKI expression skewed the above M1-like response toward an anti-inflammatory M2 state. Taken together, these findings suggest a role for QKI in restraining overt innate immune responses by regulating the Ahr/STAT1–NF-κB pathway.

## Introduction

Lipopolysaccharide (LPS), a cell wall component of Gram-negative bacteria, is one of the most powerful macrophage activators, inducing an innate immune response *via* toll-like receptor 4 (TLR4) and intracellular pathways. Overwhelming inflammatory responses are the leading cause of death in infected patients. Therefore, disclosing intracellular pathways to curb inflammatory responses against LPS stimuli is required.

Macrophages are the sentinels of the innate immune system, safeguarding against infection or tissue damage. In response to cytokines and microbial signals, they reprogram their dynamic gene expression profiles in order to produce corresponding phenotypes, referred to as M1 (classically) or M2 (alternatively) polarized macrophages. M1 macrophages are the pro-inflammatory subtype, secreting high levels of inflammatory cytokines, which are responsible for host defense against foreign pathogens, while M2 macrophages are more often associated with tissue repair and remodeling by producing anti-inflammatory mediators in order to maintain immune homeostasis (1, 2).

In fact, the two states of macrophage activation often coexist in a pathogen infection. Microbial LPS induces the activation of downstream transcription factors, such as NF-κB and signal transducer and activator of transcription 1 (STAT1), resulting in the upregulation of pro-inflammatory factors, such as TNF-α and IL-6, in macrophages (3–6). Although adequate inflammatory cytokines are essential for pathogen clearance, overproduction leads to a harmful result, such as LPS-induced shock. In order to maintain the immune balance, various mechanisms negatively regulate TLR signaling (7). Among the cellular negative regulators, the aryl hydrocarbon receptor (Ahr), acts as a transcription factor (8) and adjusts the various toxicological effects and provides immune tolerance (9, 10). Ahr is also responsible for inducing anti-inflammatory effects by negatively modulating NF-κB-dependent inflammatory responses as well as inflammasome activation in LPS-stimulated macrophages (11, 12). In addition, Ahr is involved in changing cell fate in adaptive T cell immune responses, such as the differentiation into regulatory (Treg) or T helper lineages (Th17 or Th1) (13–15). At the cytokine level, IL-10, as one of the typical alternative macrophage cytokines is able to dampen the inflammatory responses by restraining both the expression and function of TNF and IL-1 (16). Mechanistic analysis suggested that STAT3 activation is mainly involved in transcriptional regulation of various suppressive factors (17, 18). Thus, defining the intracellular molecular mechanism underlying the two macrophage polarization states will be valuable in inflammation-related disease treatments.

RNA-binding proteins are a group of proteins responsible for gene regulation at the posttranscriptional level by modulating mRNA splicing, stability, and translation (19, 20). Several proteins are known to exert key regulatory roles in immune responses. For example, myeloid cell tristetraprolin protected mice against LPS-induced septic shock through posttranscriptional regulation of TNF mRNA stability (21). In response to virus infection, a human RNA-binding protein called embryonic lethal abnormal vision drosophila-like 1 or Hu antigen R could stabilize IFN-β mRNA by binding with AU-rich sequences at its 3′ untranslated regions (22). Moreover, the RNA-binding protein, quaking (QKI), belonging to the signal transduction and activation of RNA family, displayed various critical functions in early embryonic development (23), as well as additional cellular processes (24–26). The *QKI* gene mainly encodes three protein isoforms called QKI5, QKI6, and QKI7 (27, 28). All of them contain the featured KH RNA-binding domain, but possess various lengths in their C-terminus (27, 28). QKI5, which harbors a nuclear localization signal, is the most abundant isoform and is predominantly located in the nucleus. QKI6 and QKI7 are dynamically transported between the nucleus and cytoplasmic regions and form homo or hetero-dimers with QKI5 to regulate target gene expression by specifically binding to the conserved QKI response element (QRE) on target mRNAs (29).

Recently, van der Veer et al. reported that QKI promoted monocyte differentiation toward the pro-atherosclerotic macrophage lineage, significantly altering more than 1,000 QKI-dependent mRNA levels (19). Previous findings have suggested that QKI itself precisely regulates the maturation process of monocytes to macrophages *via* negative modulation of colony-stimulating factor receptor stability (30). However, its role in the regulation of macrophage polarization, especially in host defense against microbial LPS, has yet to be documented.

We developed a myeloid-specific QKI knockout mouse model. The *in vitro* and *in vivo* data indicated that QKI deficiency made mice more susceptible to endotoxic shock, which was rescued by overexpression of QKI5 in peritoneal macrophages, favoring M2 polarization, with lower levels of pro-inflammatory cytokines. The QKI-mediated Ahr/STAT1-NF-κB pathway was involved. Therefore, targeting QKI-related signals in macrophages is a novel method to turn off the exaggerated “inflammatory storm” against LPS.

## Materials and Methods

### Cells

RAW 264.7 cells (American Type Culture Collection) were cultured in RPMI-1640 with 10% fetal bovine serum (Gibco by Life Technologies). Mouse peritoneal macrophages were obtained as described previously (31). Three days before collecting peritoneal cells, 8- to 12-week-old mice were injected with 1 ml of 3% thioglycollate medium. Cells were further enriched by discarding the culture medium containing non-adhesive cells after seeding into 6-well plates for 2 h. Bone marrow-derived macrophages were harvested as previously described (32). In brief, bone marrow cells were collected from femurs of male QKI^fl/fl^ and LysMCre QKI^fl/fl^ mice. These cells were seeded into Plastic petri dishes in complete RPMI 1640 medium that was supplemented with 20 ng/ml recombinant murine macrophage colony-stimulating factor (M-CSF, Peprotech, Rock Hill, USA) for 7 days. Adherent cells were washed with PBS and M1 macrophages were induced by 1 µg/ml LPS from *Escherichia coli* O111:B4 (Sigma-Aldrich), while M2 macrophages were obtained by adding 20 ng/ml IL-4 plus 20 ng/ml IL-13 (PEPROTECH, Rocky Hill, NJ, USA).

### Cells Counts in Peritoneal Lavage Fluid

Peritoneal lavage fluid was collected 48 h after LPS injection and placed on microscope slides by centrifugation. After fixation with methanol, slides were stained with Wright-Giemsa stain and observed under a light microscope to determine differential cell counts.

### *QKI5* Silencing or Overexpression in RAW 264.7 Cells

The construction of the shQKI5 or QKI5 overexpression plasmid and virus packaging were described in our previous study (33). In brief, RAW 264.7 cells were adjusted to the appropriate concentration so that they reached 50% confluence at the time of infection. Complete RPMI-1640 medium containing 8 µg/ml polybrene (Santa Cruz Biotechnology) was added. Then QKI5 shRNA, the QKI5 overexpression plasmid, and lentiviral particles with empty vector were added separately to different wells and mixed gently. Growth medium was then replaced after 24 h. To select transfected cells, puromycin (4 µg/ml, for siQKI5, InvivoGen) or blasticidin (2 µg/ml, for QKI5 overexpressed cells, InvivoGen) was added to the medium. The effects of silencing or overexpressing QKI in the infected cells were then tested by western blotting.

### Indirect Immunofluorescence Analysis

RAW 264.7 cells were grown on coverslips overnight. PBS-washed cells were fixed with 4% formaldehyde for 20 min and then permeabilized with 0.1% Triton X-100 for 15 min. After blocking with 5% goat serum for 1 h, cells were incubated with 1:1000 dilution of rabbit anti-mouse p65 antibody (Cell Signaling Technology, D14E12) for 30 min. After washing, cells were incubated with a 1:1000 dilution of DyLight 594 conjugated goat anti-rabbit IgG (Thermo Scientific) for 30 min and counterstained with DAPI (Sigma) to stain cell nuclei. Microscopy (Olympus IX71) was used for observing cells and cellSens imaging software was used to capture the images.

### Real-Time PCR

Total RNA was isolated and reverse transcribed to cDNA with M-MuLV reverse transcriptase (Takara). Real-time PCR was carried out with AceQ qPCR SYBR Green Master Mix (Vazyme) on the BioRad CFX96 Real-Time PCR Detection System (BioRad) with specific primers. The primer sequences were as follows: QKI (Accession number: NM_001159517.1), 5′-TAGCAGAGTACGGAAAGACATG-3′(forward) and 5′-GGGTATTCTTTTACAGGCACAT-3′(reverse); iNOS (Accession number: NM_010927.4), 5′-GCAGAGATTGGAGGCCTTGTG-3′(forward) and 5′-GGGTTGTTGCTGAACTTCCAGTC-3′(reverse); TNF-α (Accession number: NM_013693.3), 5′-GTGGAACTGGCAGAAGAGGC-3′(forward) and 5′-AGACAGAAGAGCGTGGTGGC-3′(reverse); IL-6 (Accession number: NM_031168.2), 5′-ATGGATGCTACCAAACTGGAT-3′(forward) and 5′-TGAAGGACTCTGGCTTTGTCT-3′(reverse); IL-10 (Accession number: NM_010548.2), 5′-GGGTTGCCAAGCCTTATCG-3′ (forward) and 5′-TCACTCTTCACCTGCTCCACT-3′(reverse); Arg-1 (Accession number: NM_007482.3), 5′-GGGAAGACAGCAGAGGAGGT-3′(forward) and 5′-TAGTCAGTCCCTGGCTTATGG-3′(reverse); CD206 (Accession number: NM_008625.2) 5′-GCAGGTGGTTTATGGGATGT-3′(forward) and 5′-GGGTTCAGGAGTGTTGTGG-3′(reverse); Fizz1 (Accession number: NM_020509.3), 5′-CCAATCCAGCTAACTATCCCTCC-3′(forward) and 5′-ACCCAGTAGCAGTCATCCCA-3′(reverse); Ym1 (Accession number: NM_009892.3), 5′-CAGGTCTGGCAATTCTTCTGAA-3′(forward) and 5′-GTCTTGCTCATGTGTGTAAGTGA-3′(reverse); β-actin (Accession number: NM_007393.5), 5′-GTGACGTTGACATCCGTAAAGA-3′(forward) and 5′-GCCGGACTCATCGTACTCC-3′(reverse).

### Cytokine and Alanine Aminotransferase (ALT) Assay

The concentrations of cytokines were measured by ELISA kits (Dakewe Biotech Co. Ltd, Shenzhen, China), according to the manufacturer’s instructions. The concentration of ALT was measured using the ALT assay kit (Nanjing JianCheng Bioengineering Institute).

### Flow Cytometry

For surface cell staining, 5 × 10^5^ cells were incubated for 15–20 min with anti-mouse CD16/CD32 to block non-specific binding of immunoglobulins to Fc receptors expressed on monocytes, macrophages, and granulocytes (Biolegend, clone 93). Labeling was performed for 30 min with specific antibodies. The following antibodies were used: PE anti-mouse CD11b (Biolegend, clone M1/70), FITC anti-mouse F4/80 (eBioscience, clone BM8), and FITC anti-mouse Ly6G (Biolegend, clone 1A8). All staining was done on ice in PBS supplemented with 2% BSA followed by washing once in cold PBS. The FACS Vantage system (Becton-Dickinson, San Jose, CA, USA) was used to analyze samples.

### Western Blot

Cells were lysed and centrifuged at 12,000 rpm for 15 min. After quantification of the protein concentration using a BCA kit, the same amounts of proteins were resolved on SDS polyacrylamide gels and transferred onto a PVDF membrane (Millipore). The membrane was blocked in 5% non-fat milk with TBST with 0.05% Tween for 2 h at room temperature and then incubations were performed with the indicated primary antibodies for QKI (Sigma), Ahr (R&D Systems), STAT1 and phosphor-STAT1 Tyr701 (Sangon Biotech), NF-κB p65 and phospho-NF-κB p65 (Ser536) (Cell Signaling Technology, clones D14E12 and 93H1), NLRP3 (Abcam), procaspase-1 and active caspase-1 (Abcam, clone EPR16883), and β-actin (Abcam) overnight at 4°C. After washing with TBST three times for 10 min each, the membrane was incubated with HRP-conjugated secondary antibodies for 1 h at room temperature. Next, the membrane was washed in TBST and detected by the UVP ChemiDoc-It 510 imaging system.

### Chromatin Immunoprecipitation Assay

Chromatin immunoprecipitation analysis was accomplished using the Magna ChIP™ A kit (Millipore). In brief, QKI5-silenced RAW 264.7 cells and control cells were incubated with medium or LPS (1 µg/ml) for 4 h, crosslinked in 1% formaldehyde for 10 min at room temperature and quenched with unreacted formaldehyde by the addition of 0.125 M glycine. Cells were washed twice with PBS and suspended in cell lysis buffer containing protease inhibitors, incubated on ice for 15 min, and centrifuged at 800 × *g* for 5 min. The pellet was homogenized in nuclei lysis buffer containing protease inhibitors and subjected to sonication on ice, followed by incubation overnight at 4°C with anti-Ahr (Thermo Fisher, clone RPT9), anti-p50 (Abcam), anti-p65 (Merck), anti-STAT1 (CST), normal mouse IgG, or normal rabbit IgG in the presence of protein A magnetic beads. The reactions were incubated overnight at 65°C to reverse the cross-links. DNA was purified by spin column and analyzed by PCR with the following primers: 5′-CGATGCTAAACGACGTCACATTGTGCA-3′ and 5′-CTCCAGAGCAGAATGAGCTACAGACAT-3′, specific for the κB site in the IL-6 promoter.

### RNA Immunoprecipitation

RAW 264.7 cells (1 × 10^7^) were stimulated with LPS for 6 h and harvested and re-suspended in 1.28 M sucrose, 40 mM Tris–HCl at pH 7.5, 20 mM MgCl_2_, and 4% Triton X-100 for 20 min on ice. Cells were centrifuged at 2,500 × *g* for 15 min and the cell pellet was lysed in RIP buffer (150 mM KCl, 25 mM Tris at pH 7.4, 5 mM EDTA, 0.5 mM DTT, 0.5% NP40) and centrifuged at 13,000 rpm for 10 min. Then the rabbit anti-QKI antibody (Bethyl Laboratories) or rabbit IgG (Bethyl Laboratories) was added to the supernatants and incubated for 2 h at 4°C. The protein A/G PLUS-Agarose was added and incubated for 1 h at 4°C. After washing with RIP buffer three times, the complexes were incubated with 0.5 mg/ml proteinase K at 55°C for 15 min. Trizol (Life Technologies) was added to extract the RNA. Reverse transcription was carried out and real-time PCR was performed using the following specific primers for Ahr: AACTTCAGCAGGAAAAACAGGG (forward) and ATTTACTTTAACTTCTGGGACAA (reverse). The level of β-actin mRNA in each immunoprecipitation sample was used to normalize to the RIP results.

### Mice

Heterozygous QKI-floxed transgenic (QKI^fl/wt^) mice were generated. These mice were backcrossed with C57BL/6 mice (Animal Center of the Fourth Military Medical University) for 11 generations under specific, pathogen-free conditions. Heterozygous breeding pairs were used to generate homozygote QKI^fl/fl^ mice. QKI conditional knock mice (LysMCre QKI^fl/fl^) were generated by serial breeding of QKI^fl/fl^ mice with mice that have a Cre recombinase controlled by a LysM promoter, in which the conditional QKI allele is excised in myeloid cells. Age-matched QKI^fl/fl^ and LysMCre QKI^fl/fl^ (mice were heterozygous for LysM Cre) male mice were used in all experiments. All mouse experiments and procedures were approved by the Laboratory Animal Center of Fourth Military Medical University and conducted in conformity with the ethical standards.

### LPS-Induced Endotoxin Shock

For the LPS-induced endotoxin shock model, LPS (50 mg/kg or 40 mg/kg, Sigma) was intraperitoneally (i.p.) injected into the mice. Body temperatures were monitored using a rectal thermometer at various times following LPS injection. Death of mice was recorded and the data were analyzed for statistical significance of differences between the groups. For rescue experiments, 1.2 × 10^8^ peritoneal macrophages were isolated from 20 male, C57BL/6 mice (6–8 weeks old) as previously described and divided into cell culture dishes (6.0 cm). The cells were transduced by either QKI5 overexpression plasmid or by lentiviral particles with empty vector and incubated at 37°C, in a 5% CO_2_ incubator for 8 h. Polybrene (8 µg/ml) was supplemented to enhance the infection efficiency. Cells were washed and maintained in complete medium followed by blasticidin (4 µg/ml) selection for 3 days. Peritoneal macrophages overexpressing QKI5 (2.8 × 10^7^ cells) were collected and i.p. injected into mice (2 × 10^6^ cells per mouse) 0.5 h after LPS injection (40 mg/kg). The survival was recorded and the serum was collected.

### Statistical Analysis

Statistical analyses were performed using the Student’s *t*-test (two comparisons, or two-tailed) and one-way ANOVA (multiple comparisons). For the survival studies, the log-rank test was used to determine significance*. p* < 0.05 was deemed significant.

## Results

### Dynamic Changes in QKI Expression in Differently Polarized Macrophages

**Figure 1:**
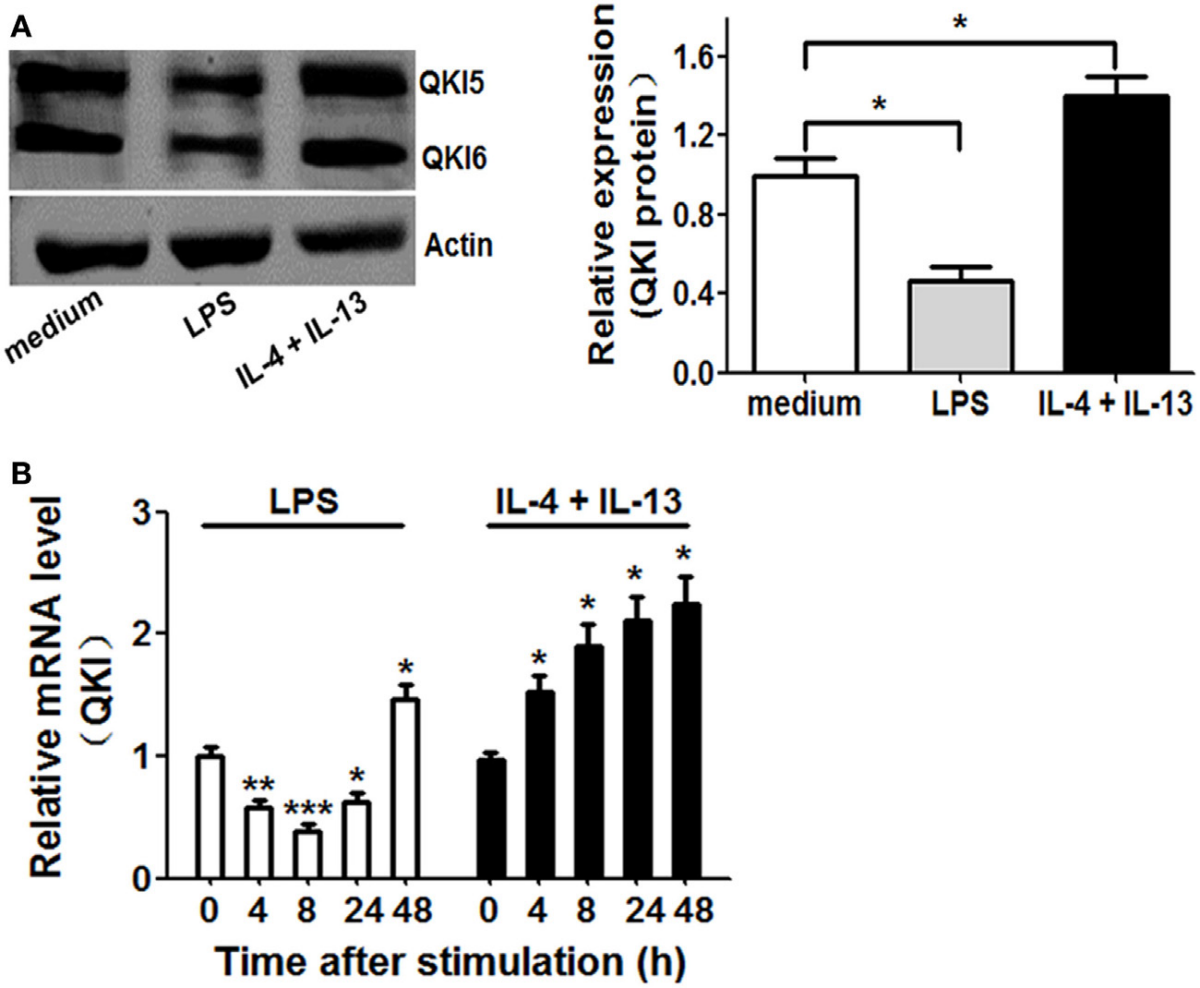
Expression of quaking (QKI) in differently polarized macrophages. **(A)** Peritoneal macrophages from wild-type (WT) mice were stimulated with 1 µg/ml lipopolysaccharide (LPS) or IL-4 (20 ng/ml) plus IL-13 (20 ng/ml) for 16 h. Total cell lysates were subjected to western blot to analyze the protein expression of QKI, with actin as an internal control. One representative immunoblot is shown (on the left). Graphs (on the right) are representative quantification of the band intensity for immunoblots from three independent experiments. **(B)** Real-time PCR analysis of QKI mRNA in mouse macrophage RAW 264.7 cells left untreated or stimulated for 4, 8, 24, or 48 h with 1 µg/ml LPS or IL-4 (20 ng/ml) plus IL-13 (20 ng/ml). Results presented relative to those of untreated macrophages, set as 1. Actin served as an internal control. All bars represent the mean of measurements from three independent experiments, and the error bars indicate ±SEM. *p < 0.05, **p < 0.01, ***p < 0.001.

To determine whether QKI is involved in macrophage polarization, we first analyzed QKI protein expression in mouse peritoneal macrophages at 16 h after stimulation with M1 (LPS) or M2 (IL-4 plus IL-13) polarizing agents. As shown in Figure 1A, QKI protein expression was reduced by LPS and increased by IL-4 plus IL-13 treatment. QKI mRNA expression was determined using primers specific for a common sequence that can detect QKI5, QKI6, and QKI7. In RAW 264.7 cells, mRNA expression showed biphasic patterns in M1 macrophages, showing a decrease by 2.5-fold within 8 h, while beyond 24 h, transcripts progressively returned to their initial level and then increased again at 48 h. However, under M2 polarization conditions, QKI expression increased above twofold at 48 h (Figure 1B). These results suggest that QKI expression is closely related to the macrophage polarization state, potentially participating in the establishment of macrophage plasticity.

### Higher QKI Levels Causes a Shift of Macrophages from M1 Toward M2

**Figure 2:**
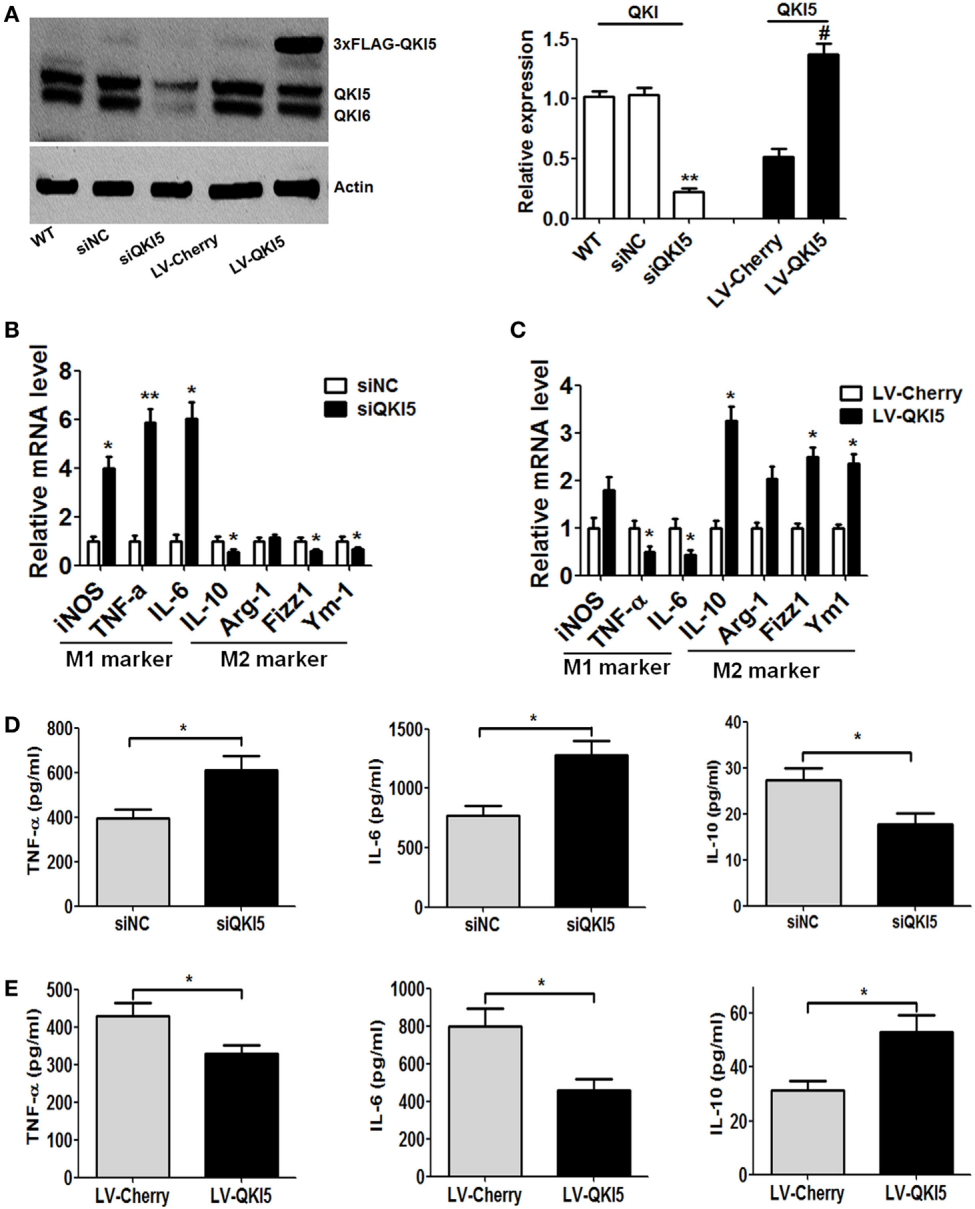
Dysregulation of quaking (QKI) expression influenced the production of macrophage lineage-specific cytokines. **(A)** Western blots were performed to determine the effects of QKI knockdown (siQKI5 and siNC was a scramble control) or overexpression (LV-QKI5, and LV-Cherry was a control) in RAW 264.7 cells using an anti-QKI antibody. One representative immunoblot is shown (on the left) and the graph (on the right) presents quantification of the band intensity for immunoblots from three independent experiments. **p < 0.01 versus WT control; #*p* < 0.05 versus LV-Cherry group. **(B,C)** Real-time PCR analysis of relative mRNA expression of the M1 markers (iNOS, TNF-α, and IL-6) and M2 markers (IL-10, Arg-1, Fizz1, and Ym1) by QKI silencing **(B)** or overexpression **(C)** RAW 264.7 cells stimulated with 1 µg/ml lipopolysaccharide (LPS) for 16 h. **(D,E)** Enzyme-linked immunosorbent assay (ELISA) of the production of TNF-α, IL-6, and IL-10 by QKI-silenced **(D)** or overexpressed **(E)** RAW 264.7 cells stimulated for 24 h with 1 µg/ml LPS. All the bars represent the mean of measurements from three independent experiments, and the error bars indicate ±SEM. *p < 0.05, **p < 0.01.

Following the above observations, we continued to explore whether QKI could directly affect the expression of M1/M2 macrophage phenotypic markers. QKI5 silencing or overexpression in RAW 264.7 cells was achieved. Western blot detection confirmed the efficacy of QKI5 dysregulation (Figure 2A). Interestingly, we observed that silencing QKI5 could also interfere with the expression of QKI6 and QKI7 in macrophages (unpublished data). After LPS stimulation, significant mRNA expression of iNOS, TNF-α, and IL-6 was observed, while reduced levels of anti-inflammatory factors such as IL-10, Fizz1, and Ym1 were seen in QKI5-silenced cells (Figure 2B). ELISA further confirmed the above changes at the protein level (Figure 2D). On the contrary, QKI5 overexpression led to decreased levels of TNF-α and IL-6 but increased levels of IL-10, Fizz1, and Ym1 (Figure 2C). Secretions of TNF-α and IL-6 were also reduced (Figure 2E). Production of anti-inflammatory IL-10 by RAW 264.7 cells overexpressing QKI5 was enhanced, even under M1 polarization conditions (Figure 2E). Together, these results indicate that QKI5 is able to regulate macrophage polarization and skew the differentiation of macrophages toward an anti-inflammatory M2 phenotype.

### QKI Inhibits NF-κB-Mediated Inflammatory Cytokine Production in M1 Macrophages

**Figure 3:**
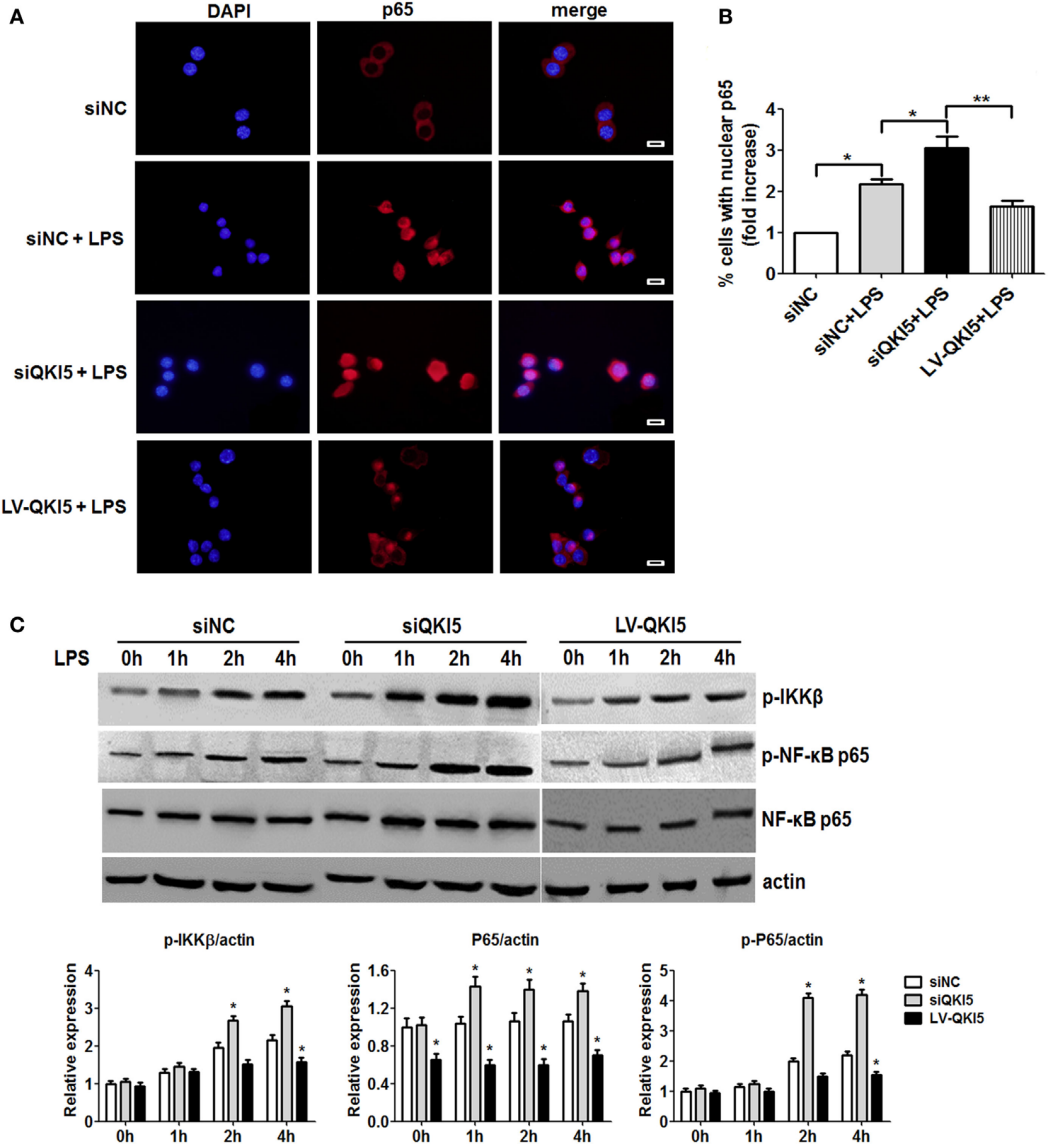
Lack of quaking (QKI)-enhanced lipopolysaccharide (LPS)-induced nuclear translocation and phosphorylation of NF-κB. **(A)** QKI-silenced or overexpressed RAW 264.7 cells were treated with 1 µg/ml LPS for 2 h. Immunostaining for p65 (red) was performed. DAPI staining is shown in blue. Scale bar, 20 µm. **(B)** Graphs show the percentage of macrophages with nuclear p65 (fold increase) in **(A)**. Briefly, 100 randomly chosen cells were counted and categorized into two groups according to the subcellular distribution of p65, including nuclear or cytoplasmic. **(C)** QKI-silenced or overexpressed RAW 264.7 cells were treated with 1 µg/ml LPS for the time indicated. One representative western blot of phospho-IKKβ, phospho-NF-κB p65, and total NF-κB p65 is shown. All the bars represent the mean of measurements from three independent experiments, and the error bars indicate ±SEM. *p < 0.05, **p < 0.01 versus siNC control.

We next sought to identify the molecular mechanism for QKI-mediated regulation of macrophage plasticity. NF-κB is the key transcriptional factor in mediating production of inflammatory cytokines, such as TNF-α and IL-6 in response to LPS stimulation. It was speculated that QKI deficiency might enhance the activation of NF-κB. While LPS-treated RAW 264.7 cells induced nuclear translocation of p65, this was significantly enhanced in siQKI5 cells (Figures 3A,B). Western blot results revealed that the phosphorylation of p65 (Ser 536) and IΚKβ and total p65 expression were significantly increased in QKI5-silenced cells compared with those in silenced control cells (siNC) after LPS treatment. By contrast, QKI5 overexpression significantly suppressed these effects (Figure 3C). Therefore, our data disclose a suppressive role for QKI5 in the LPS-induced production of pro-inflammatory cytokines by inhibiting NF-κB activation.

### Inhibition of NF-κB Transcriptional Activity and Inflammasome Activation by QKI Is Ahr-Dependent

**Figure 4:**
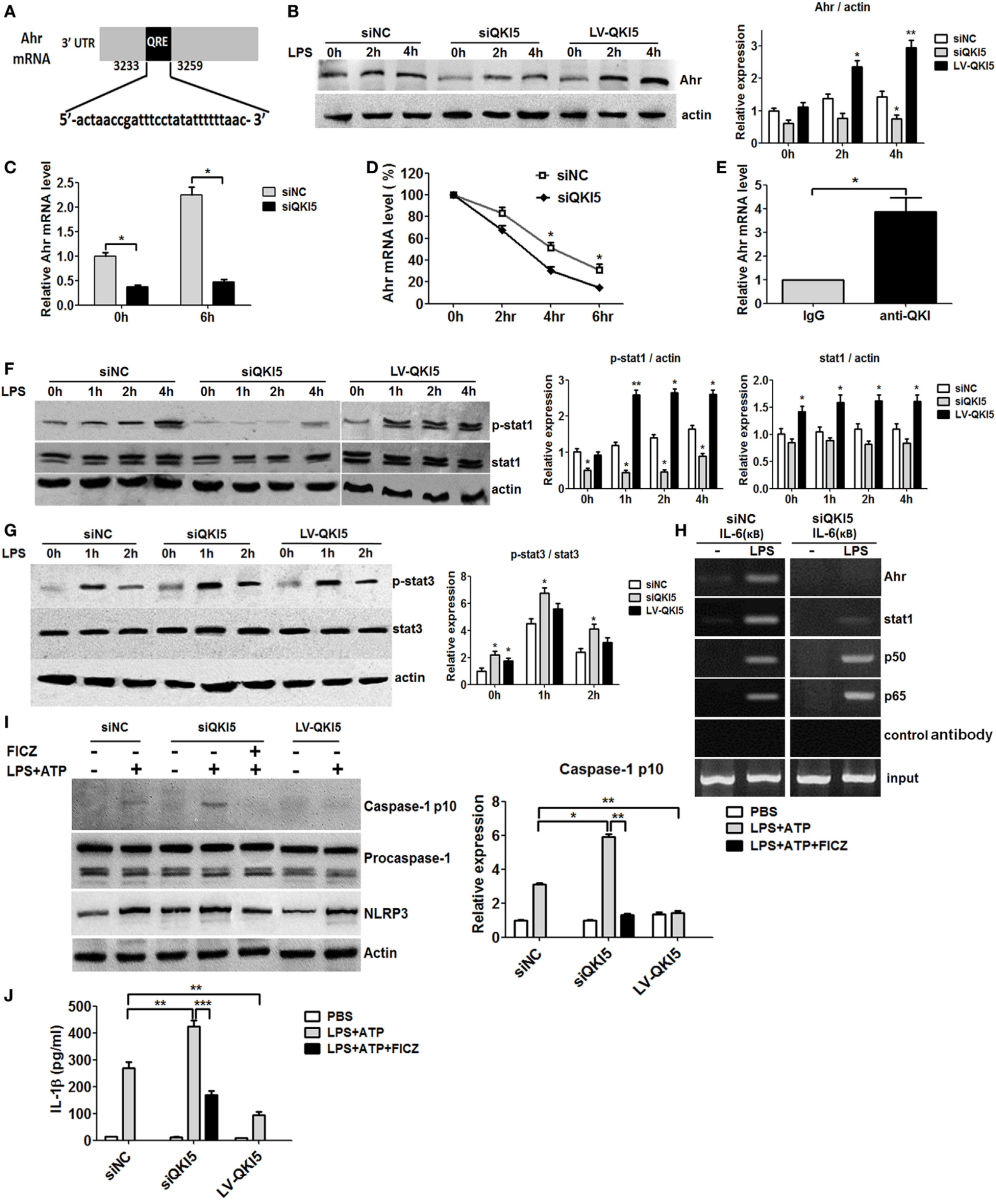
Inhibition of NF-κB transcriptional activity and inflammasome activation by quaking (QKI) was aryl hydrocarbon receptor (Ahr) dependent. **(A)** Bioinformatics analysis confirmed the QKI binding site in the 3′UTR of Ahr mRNA. Regions corresponding to a potential QKI response element (QRE) sequence are shown in shaded boxes. **(B)** QKI-silenced or overexpressed RAW 264.7 cells were stimulated with 1 µg/ml lipopolysaccharide (LPS) for the time indicated and subjected to western blot analysis. One representative immunoblot of Ahr is shown and the graph presents quantification of the band intensity for immunoblots from three independent experiments. **(C)** SiQKI5 or siNC cells were stimulated with or without 1 µg/ml LPS for the time indicated. The Ahr mRNA level was analyzed by real-time PCR. **(D)** The decay of Ahr mRNA in QKI knockdown or control RAW 264.7 cells. Six hours after LPS (1 µg/ml) stimulation, cells were washed with PBS for four times and incubated with new medium in the presence of actinomycin D to block transcription before isolating total RNA at the indicated time points of transcription inhibition. Real-time PCR was performed for the analysis of Ahr mRNA expression. *p < 0.05 versus scramble control. **(E)** RNA immunoprecipitation analysis using QKI antibody followed by real-time PCR to measure the mRNA levels of Ahr in RAW 264.7 macrophages treated by LPS for 6 h, with β-actin used as a normalization control. **(F,G)** Cells were stimulated with 1 µg/ml LPS for the time indicated and cell lysates were analyzed by immunoblotting with specific antibodies. One representative western blot of p-STAT1, total STAT1 **(F)**, STAT3 and p-STAT3 **(G)** is shown. Graphs present the quantification of band intensity for immunoblotting from three independent experiments. **(H)** Cells were treated with 1 µg/ml LPS for 4 h and the ChIP assay was performed using anti-p65, anti-p50, anti-STAT1, and anti-Ahr antibodies. Purified DNA fragments were amplified using primers specific for the IL-6 promoter. One representative result from three independent experiments is shown. **(I)** Cells were pretreated with dimethyl sulfoxide or FICZ for 40 min and stimulated with LPS for 8 h and ATP for the last 30 min. Cell lysates were subjected to western blot analysis. One representative immunoblot of cleaved caspase-1 (p10), procaspase-1, and NLRP3 is shown. **(J)** Cells were treated as described in **(I)** and the levels of IL-1β in the culture supernatants were determined by enzyme-linked immunosorbent assay. All the bars represent the mean of measurements from three independent experiments, and the error bars indicate ±SEM. *p < 0.05, **p < 0.01.

In order to validate the signaling pathway, we investigated how QKI regulates NF-κB-mediated pro-inflammatory signaling. Bioinformatics analysis predicted that the 3′UTR region of Ahr mRNA contained one QRE motif (Figure 4A). The ablation of QKI reduced both mRNA and protein expression of Ahr, as expected (Figures 4B,C). Notably, QKI5 silencing could decrease Ahr mRNA expression in untreated RAW 264.7 cells. QKI loss led to faster degradation of Ahr mRNA, with a half-life of approximately 2.5 h (Figure 4D). To further clarify if QKI modulated Ahr expression, RIP analysis was performed to determine if QKI binds directly to Ahr mRNA. Our results revealed that the anti-QKI antibody specifically interacted with Ahr mRNA compared to control IgG (Figure 4E), indicating that Ahr mRNA stability in macrophages was directly regulated by QKI. A previous study reported that Ahr interacted with STAT1 to negatively regulate NF-κB-dependent pro-inflammatory cytokine production in response to LPS (12). Interestingly, we found that silencing QKI5 repressed the expression and phosphorylation of STAT1 (Figure 4F). Since STAT3 is closely related to LPS-induced macrophage polarization, its expression was also detected. As shown in Figure 4G, silencing QKI5 significantly promoted STAT3 activation compared with that in siNC cells, while there was no difference between QKI5-silenced and -overexpressed cells. The CHIP assay further showed that Ahr and STAT1 were recruited to the IL-6 promoter after LPS stimulation in siNC cells, but not in siQKI5 cells (Figure 4H). These results support that QKI represses LPS-induced pro-inflammatory cytokine secretion in macrophages by enhancing Ahr expression and STAT1 expression and phosphorylation, leading to the inhibition of NF-κB transcriptional activity through their promoter regions.

The inflammasome was recently reported to play a crucial role in inflammation in response to pathogens and Ahr is known to negatively regulate NLRP3 inflammasome activity, and subsequent IL-1β secretion (11). Here, we further analyzed the function of QKI on inflammasome activation. As shown in Figures 4I,J, QKI silencing resulted in increased caspase-1 cleavage and IL-1β secretion in LPS-primed macrophages treated by ATP, whereas overexpression of QKI5 had an opposite effect. However, Ahr activation by its ligand, 6-formylindolo(3,2-b)carbazole (FICZ), suppressed the enhancement of active caspase-1 and IL-1β production in QKI5-silenced macrophages. In short, our results demonstrate that QKI5 suppresses NLRP3 inflammasome activation and IL-1β expression, partially in an Ahr-dependent manner.

### Generation of Macrophage-Specific *QKI*-Deficient Mice

**Figure 5:**
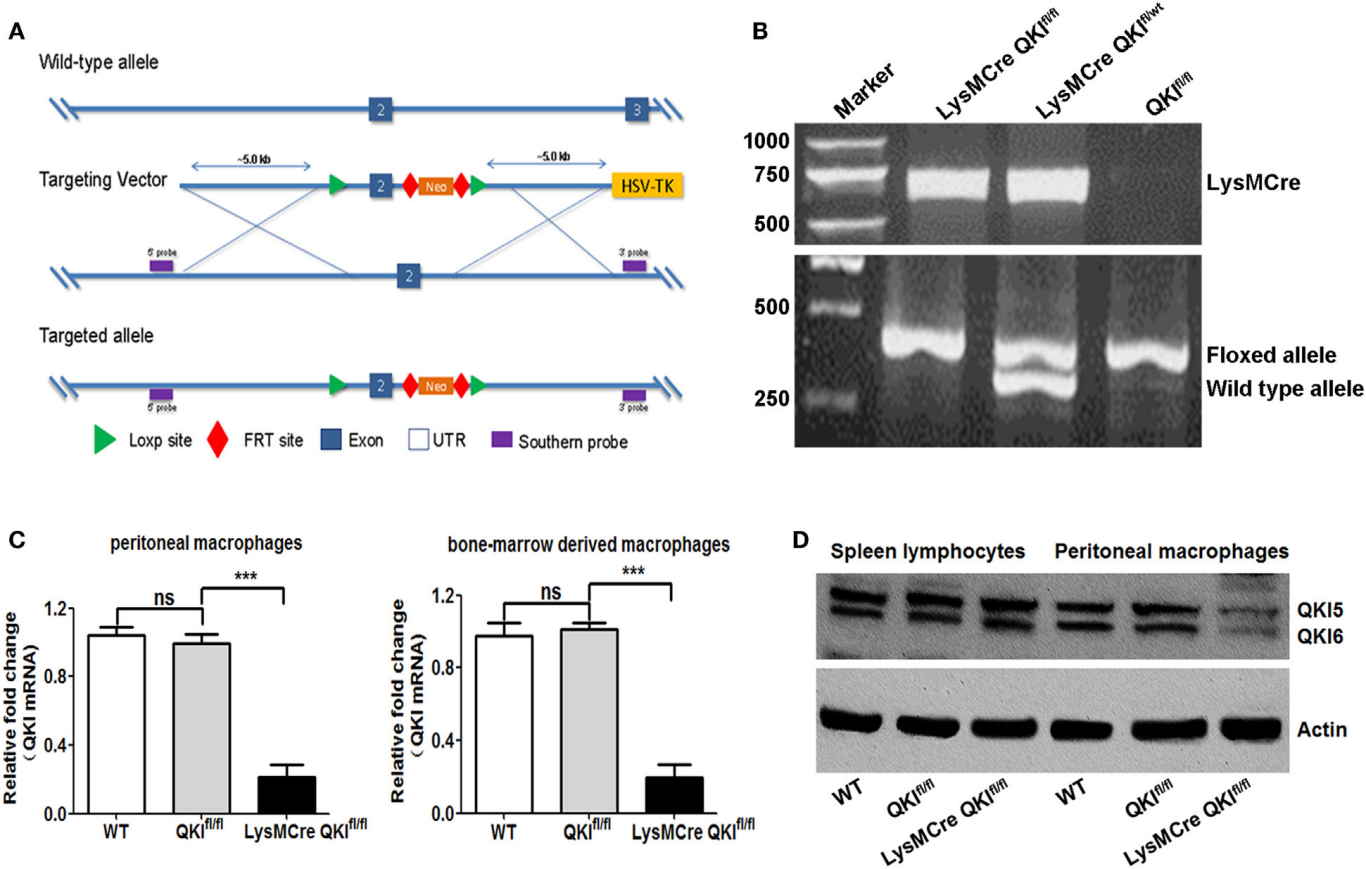
Generation and characterization of myeloid-specific quaking (QKI)-deficient mice. **(A)** The targeting strategy of QKI LoxP mice is shown. **(B)** Total genomic DNA from mouse tail tissues was isolated and subjected to PCR analysis using a site-specific primer. The 700-bp product indicates the presence of the cre allele. The 410-bp product indicates the presence of the QKI-floxed allele, while the 370-bp product indicates the presence of the wild-type (WT) allele. **(C)** WT, QKI^fl/fl^, and LysMCre QKI^fl/fl^ mouse thioglycollate-induced peritoneal macrophages and bone marrow-derived macrophages were analyzed for QKI mRNA expression by quantitative PCR. Actin was used as the housekeeping gene. Levels of QKI in WT mice were set as one. Each group included 10 mice. All the bars represent the mean of measurements from three independent experiments, and the error bars indicate ±SEM. **(D)** Peritoneal macrophages and spleen lymphocytes from WT, QKI^fl/fl^, and LysMCre QKI^fl/fl^ mouse were isolated and lysed. Total cell lysates were subjected to western blot analysis using rabbit polyclonal anti-QKI antibody. Actin was used as the internal control. ***p < 0.001.

To explore the *in vivo* functions of QKI, we employed a conditional gene targeting strategy to generate QKI-floxed mice by introducing two loxP sites flanking exon 2 of the quaking gene (QKI^fl/fl^) (shown in Figure 5A). The QKI-floxed mice were mated to mice expressing the Flp recombinase to delete the *neo* gene. Then these mice were crossed with LysM Cre mice. In the resulting mice, quaking gene expression was specifically inactivated in myeloid cells, referred to as LysMCre QKI^fl/fl^ mice. The genotypes of mice were identified by PCR using three different types of PCR primers and mouse tail DNA as the template. Consequently, the 700-bp amplicon was indicated as Cre allele-positive mice, the 410-bp product indicated the presence of the floxed allele, and the 370-bp product indicated the presence of the WT allele (Figure 5B). Myeloid cell-specific deficiency of QKI in LysMCre QKI^fl/fl^ mice was verified by the reduced expression of QKI at both mRNA and protein levels in peritoneal macrophage cells, compared to that in spleen lymphocytes (Figures 5C,D, *p* < 0.001). The WT and QKI^fl/fl^ mice were used as controls. For all experiments, mice were heterozygous for LyM Cre.

### QKI-Deficient Mice Are Hyper-Responsive to LPS-Induced Endotoxic Shock

**Figure 6:**
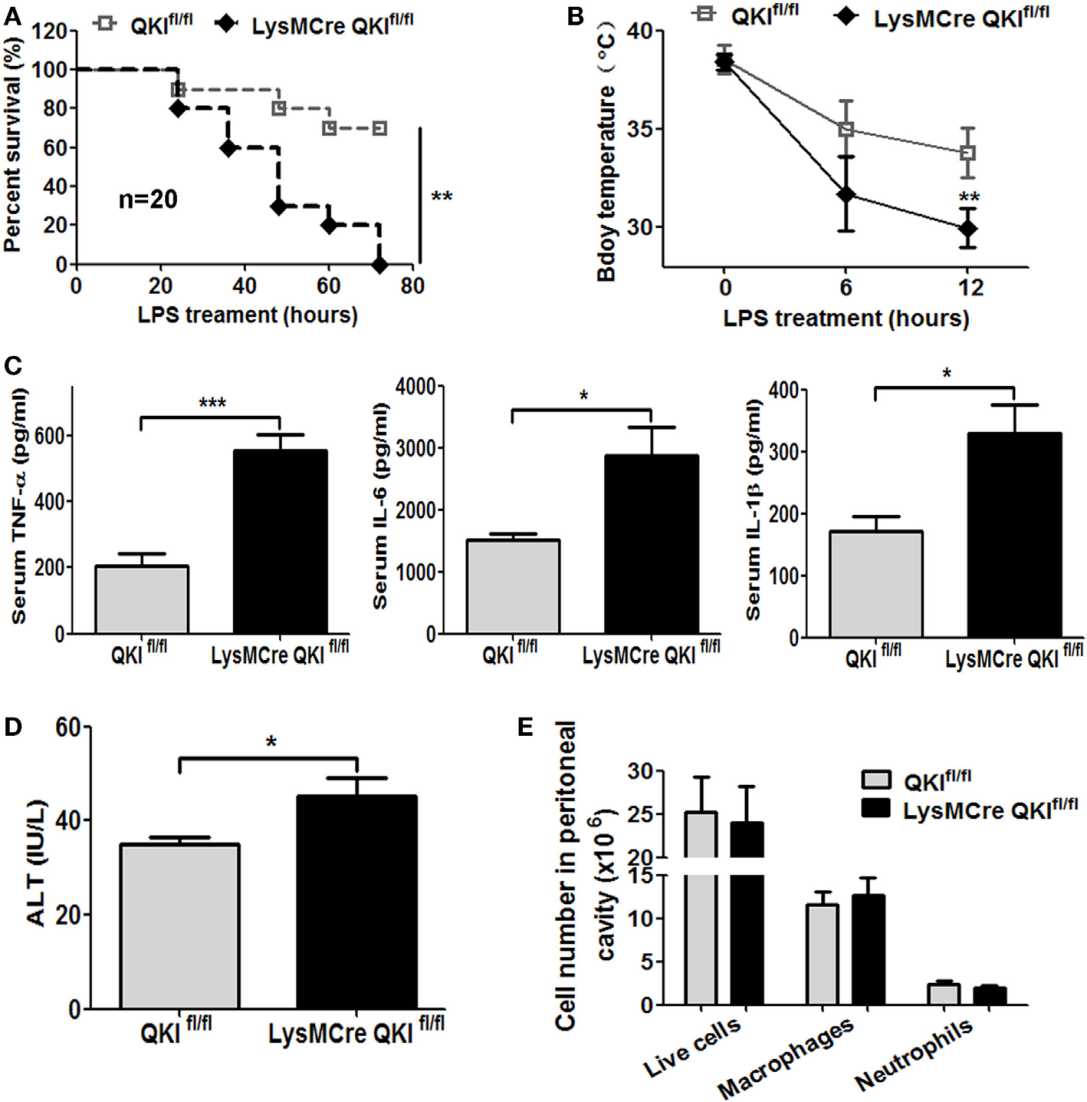
Macrophage-specific quaking (QKI) deficiency caused high susceptibility to endotoxic shock. **(A)** Age- and sex-matched QKI^fl/fl^ and LysMcre QKI^fl/fl^ mice were intraperitoneally injected with 50 mg/kg lipopolysaccharide (LPS). The survival was documented. The data shown are pooled from the two independent experiments (*n* = 20). **(B)** Body temperature of QKI^fl/fl^ and LysMCre QKI^fl/fl^ mice LPS treatment and 6 h and 12 h after LPS injection was measured using a rectal thermometer. **(C)** QKI^fl/fl^ and LysMCre QKI^fl/fl^ mice were challenged with LPS. Serum was obtained 4 h after LPS injection. Inflammatory cytokines were analyzed by using the Dakawe mouse enzyme-linked immunosorbent assay (ELISA) kit. **(D)** QKI^fl/fl^ and LysMCre QKI^fl/fl^ mice were challenged with LPS. After 4 h, the serum of mice was obtained. Alanine aminotransferase (ALT) was tested to determine the extent of liver injury using the ALT assay kit. **(E)** Cells isolated from the mouse peritoneum 48 h post-LPS injection were stained with Wright-Giemsa. The number of total live cells, macrophages, and neutrophils were calculated. All the experiments were performed twice, independently and each time 10 mice/group were used in **(A–D)**. *n* = 20. In **(E)**, 6 mice/group were used. Data are presented as mean ± SEM. *p < 0.05, **p < 0.01, ***p < 0.001.

Using the above mice, we observed the *in vivo* function of myeloid QKI in the LPS-induced endotoxic shock model. LPS was intraperitoneally injected into mice and the survival status was observed for 72 h. As shown in Figure 6A, 70% of the QKI^fl/fl^ mice were still alive, while 100% of the LysMCre QKI^fl/fl^ mice were dead. In addition, the LysMCre QKI^fl/fl^ mice showed hypothermia, with body temperatures decreasing more rapidly than the control mice (Figure 6B, *p* < 0.01). Compared with QKI^fl/fl^, the serum levels of TNF-α, IL-1β, and IL-6 were increased significantly in LysMCre QKI^fl/fl^ mice (Figure 6C, *p* < 0.05). Since the liver is the most sensitive to infections, the levels of ALT were measured to reflect the extent of liver injury. LPS-treated mice displayed higher levels of ALT in the serum compared with serum from healthy C57BL/6 mice (about 20 U/L) (34). Moreover, as shown in Figure 6D, LysMCre QKI^fl/fl^ mice displayed much higher levels of ALT in the serum than QKI^fl/fl^ mice, indicating more severe liver injury. Cell type differentials were determined in peritoneal lavage fluid (total live cells, macrophages, and neutrophils) using Wright-Giemsa staining and were similar in the two groups of mice (Figure 6E).

### Transfer of Peritoneal Macrophages Overexpressing QKI5 Confers Resistance to LPS-Induced Endotoxic Shock in LysMCre QKI^fl/fl^ Mice

**Figure 7:**
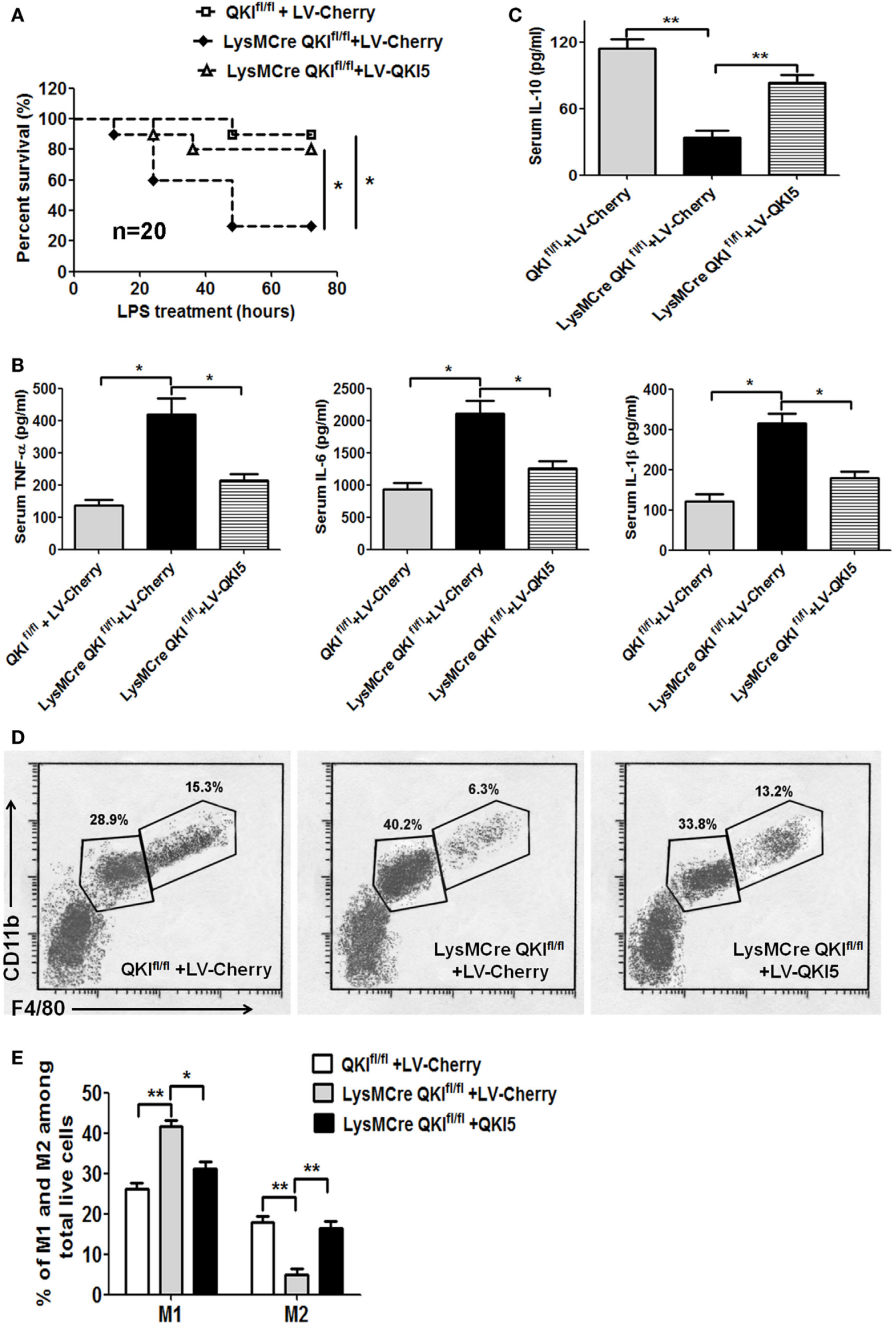
Transfer of peritoneal macrophages overexpressing QKI5 rescued lipopolysaccharide (LPS)-induced endotoxic shock. **(A)** Peritoneal macrophages overexpressing QKI5 (2 × 10^6^/mouse) or control cells (LV-Cherry) were intraperitoneally injected 0.5 h after QKI^fl/fl^ and LysMCre QKI^fl/fl^ mice were challenged with LPS (40 mg/kg). Survival was observed. The data shown are pooled from the two independent experiments (*n* = 20). **(B,C)** Serum samples were collected 4 h after LPS injection. Inflammatory cytokines TNF-α, IL-6, IL-1β **(B)**, and IL-10 **(C)** were analyzed using the Dakawe mouse enzyme-linked immunosorbent assay (ELISA) assay kit. **(D)** Twenty-four hours after LPS challenge, mouse peritoneal cells were collected and analyzed by flow cytometry to discriminate macrophage subtypes. F4/80^high^CD11b^high^ (M2) and F4/80^int^CD11b^int^ (M1) gated out of total live cells. **(E)** Quantification of M1 and M2 cells in **(D)** obtained from LPS-challenged mice. All animal experiments were performed twice, independently and each time 10 mice/group were used in **(A–C)**. *n* = 20. For **(D)**, 6–9 mice/group were used. Data are presented as mean ± SEM. *p < 0.05, **p < 0.01, ***p < 0.001.

Since the conditional QKI-deficient mice were more sensitive to endotoxin challenge, we further validated this by restoring QKI5 expression in peritoneal macrophages to determine if this would rescue the lethal phenotype. Peritoneal macrophages, with a purity of >90% and isolated from C57BL/6 mice, were used to construct macrophages either overexpressing QKI5 or control virus. The efficacy of QKI5 overexpression in peritoneal macrophages was then verified by western blot analysis. Our results showed that the QKI5 protein expression increased twofold in LV-QKI5 cells compared with that in wild-type (WT) or LV-Cherry cells (data not shown). These cells were transferred into LPS-challenged LysMCre QKI^fl/fl^ mice, leading to significantly improved survival, from 30 to 80% (Figure 7A, *p* < 0.05). This corresponded to a decrease in pro-inflammatory mediators in the serum, including TNF-α, IL-6, and IL-1β (Figure 7B, *p* < 0.05), while there was an increase in anti-inflammatory cytokines, such as IL-10 (Figure 7C, *p* < 0.01).

Given the reduced pro-inflammatory responses in LysMCre QKI^fl/fl^ mice after transferring peritoneal macrophages that overexpress QKI5 in the LPS-induced mice model, alternatively activated macrophages (M2) were anticipated. Thus, we measured changes in the proportion of peritoneal M1 macrophages (F4/80^int^CD11b^int^) and M2 macrophages (F4/80^hi^CD11b^hi^) (35). Compared to macrophages in QKI^fl/fl^ mice, an increase in M1 macrophages and a decrease in M2 macrophages were shown in LPS-treated LysMCre QKI^fl/fl^ mice transferred with control cells. However, the proportion of M2 macrophages in the peritoneal cavity of LysMCre QKI^fl/fl^ mice after transfer of peritoneal macrophages overexpressing QKI5 was significantly elevated, with a parallel reduction in M1 macrophages (Figures 7D,E). Interestingly, transferring peritoneal macrophages that were derived from WT mice and transfected with LV-cherry did not improve the lethal state of QKI-floxed mice after LPS treatment. We anticipated that endogenous QKI expression would be inhibited after transferring into LPS-stimulated mice under a state of M1 polarization. However, QKI was still overexpressed in exogenous macrophages that were still present, exerting an anti-inflammatory effect.

## Discussion

In response to the invasion of bacteria and other types of pathogens, macrophages, the main innate immune cells, phagocytize pathogens and secrete a variety of pro-inflammatory cytokines, which evoke the host defense responses (36). During this process, macrophage activation is dynamically regulated and diverse phenotypes often coexist (35).

Two extreme states of macrophage activation, M1 versus M2, are often described. Briefly, M1 macrophages are referred to as the pro-inflammatory subtype, secreting more inflammatory factors, such as TNF-α, IL-6, and IL-1β, exerting pathogen killing effects by enhanced phagocytosis and intracellular killing, as well as other paracrine cytokines effects (37). M2 macrophages are more anti-inflammatory and work to shut down the overt inflammatory response (32). Homeostasis of these two polarized states in acute immune responses is particularly beneficial for the body’s defense system. In clinics, either an overwhelming inflammatory state or immune suppression related to chronic inflammation is the etiologies that lead to lethal conditions (38). Therefore, understanding the intrinsic mechanisms underlying macrophage polarization will help us to gain control of innate immune responses during the development of sepsis.

It has been reported that in the LPS-stimulated THP-1 pro-monocyte activation model, there is a sequential reprogramming of metabolic and inflammatory-related signals, resulting in a switch from M1 macrophages to an M2 phenotype. The dynamic conversions between the two phenotypes are known to be mediated by several critical factors. Generally, M1 macrophages are dependent on glycolysis and hypoxia-inducible factor 1 (HIF-1α) expression, resulting in higher transcription of TNF-α and Rel-B. Subsequent skirting 1 (SirT1) elevation is responsible for promoting the M2 state by enhancing fatty acid uptake and oxidation (39). Thus, clarifying the underlying mechanisms by which the M2 state was established after M1 suppression will be valuable for the control of sepsis.

Here, we first defined a dynamic expression pattern of QKI in macrophages treated with M1 or M2 stimuli. They showed a reduced M1 state, but an enhanced M2 state, suggesting the potential role of QKI in the M2-related phenotype. Further mechanistic characterization revealed that silencing QKI led to increased expression of M1-related pro-inflammatory genes by enhancing p65 expression and phosphorylation, with decreased STAT1 phosphorylation. Whereas elevated QKI5 expression exerted an anti-inflammatory effect by upregulating the levels of IL-10 and other M2-related genes, such as Arg-1, Fizz1, and Ym1. Unexpectedly, SirT1 expression had little change following QKI alterations (data not shown). Collectively, these *in vitro* data indicated that QKI plays a dual role in modulating M1/M2 polarized states, specifically promoting M2 polarization at the expense of the M1 state. In terms of the mechanism, QKI may mediate its inflammatory suppressive effect through influence on more than one factor.

Aryl hydrocarbon receptor, a ligand-activated transcriptional factor, displays an important regulatory function in various biological processes, including detoxification and immune responses. It is also involved in LPS-induced immune tolerance in macrophages (11) by suppressing NLRP3 inflammasome transcription, downstream caspase-1 activation, and subsequent IL-1 β secretion.

In our study, we defined a novel pathway to regulate Ahr expression at the posttranscriptional level. As an RNA-binding protein, QKI affects Ahr mRNA stability by directly binding to its 3′UTR. More importantly, p65-mediated transcriptional activity at the promoter region of target genes is also prohibited by QKI-mediated Ahr changes. In addition, the alterations in the NLRP3 inflammasome activation, caspase-1 cleavage, and IL-1β secretion are also alleviated by Ahr signaling in QKI-overexpressed cells. Therefore, these findings confirm the importance of Ahr signaling in mediating QKI-related anti-inflammatory regulation.

It is worth mentioning that consistent with our observations, another group has also reported that miR-155-mediated downregulation of QKI in RAW 264.7 cells was important for enhanced responses to LPS, as well as for leukemogenesis (40). Moreover, they defined that the c-Jun N-terminal kinase was involved in QKI-induced anti-inflammatory responses, using the p38 MAPK pathway. However, they did not show the effects by using primary cells or in an *in vivo* mouse model.

In order to test the *in vivo* function of QKI, we established myeloid-specific QKI knockout mice. The overall inflammatory responses in the LPS-induced endotoxic shock model were more severe in conditional QKI-deficient mice, parallel with higher levels of TNF-α, IL-6, and IL-1β. The LPS-induced lethal phenotype in QKI-deficient mice could be largely alleviated by transferring peritoneal macrophages overexpressing QKI5, which was associated with decreased production of the systemic pro-inflammatory cytokines but increased numbers of M2 macrophages and higher levels of IL-10. Strikingly, the peritoneal macrophage subgroup analysis revealed that transfer of macrophages overexpressing QKI5 significantly induced a shift of M1 (F4/80^int^CD11b^int^) macrophages toward the M2 (F4/80^hi^CD11b^hi^) phenotype.

Compared to monocytes, neutrophils have a shorter lifespan and play a significant role in the rapid removal of microorganisms. Granulocyte homeostasis constitutes a subtle balance between being helpful or harmful in terms of damage to the tissues in response to microbes (41). Our current mouse model features QKI deficiency in the myeloid lineage. Following LPS treatment, the numbers of macrophages are mildly elevated whereas neutrophils are slightly decreased, with no significant differences in either cell type. Since we did not perform any relevant functional tests in QKI-deficient neutrophils, it is difficult to draw a definitive conclusion on whether the lethal phenotype with exaggerated inflammatory responses in LysMCre QKI^fl/fl^ mice against LPS is related to neutrophils or not. Therefore, more experimental evidence is needed in this area.

On the whole, our results suggest a novel role for QKI in restraining LPS-induced overt innate immune responses in mice by favoring the anti-inflammatory M2-polarized macrophages rather than the pro-inflammatory M1-polarized macrophages. Therefore, modulating the QKI expression level may be a potential way to treat macrophage-mediated inflammatory diseases such as sepsis.

## Ethics Statement

This study was carried out in accordance with the recommendations of the Laboratory Animal Center of Fourth Military Medical University.

## Author Contributions

Conceived and designed the experiments: LW, J-LY, Z-FL, and D-SZ; performed the experiments: LW, D-SZ, B-JR, C-MX, Z-CY, H-YL, Y-HJ, Z-YW, AX, and YY; analyzed the data and wrote the paper: LW, J-LY, B-JR, and Z-FL.

## Conflict of Interest Statement

The authors declare that the research was conducted in the absence of any commercial or financial relationships that could be construed as a potential conflict of interest.
